# High molecular weight Intraarticular hyaluronic acid for the treatment of knee osteoarthritis: a network meta-analysis

**DOI:** 10.1186/s12891-020-03729-w

**Published:** 2020-10-23

**Authors:** Charles D. Hummer, Felix Angst, Wilson Ngai, Craig Whittington, Sophie S. Yoon, Lionel Duarte, Colleen Manitt, Emil Schemitsch

**Affiliations:** 1 Premier Orthopaedics and Sports Medicine, 300 Evergreen Drive, Suite 200, Glen Mills, PA 19342 USA; 2 Research Department, Rehabilitation Clinic (‘RehaClinic’), Bad Zurzach, Switzerland; 3 Sanofi, Global Medical, Bridgewater, NJ USA; 4 Doctor Evidence, Santa Monica, CA USA; 5 International Centre for Professional Development in Health and Medicine, Québec, Canada; 6 London Health Sciences Centre, London, Ontario Canada

**Keywords:** Hyaluronic acid, Knee osteoarthritis, Molecular weight, Meta-analysis

## Abstract

**Background:**

The 2013 American Academy of Orthopaedic Surgeons (AAOS) guidelines made strong recommendations against intraarticular hyaluronic acid (IAHA) for patients with knee osteoarthritis (OA), as evidence supporting improvements in pain did not meet the minimal clinically important improvement (MCII) threshold. However, there may be important distinctions based on IAHA molecular weight (MW). Hence our objective was to evaluate the efficacy of IAHAs in knee OA based on molecular weight.

**Methods:**

Randomized controlled trials were searched within MEDLINE, Embase, and CENTRAL and selected based on AAOS criteria. A pain measure hierarchy and longest follow-up were used to select one effect size from each trial. Mean differences between interventions were converted to standardized mean differences (SMDs) and incorporated into a random-effects Bayesian network meta-analysis. High MW (HMW) was defined as ≥6000 kDa, and low MW (LMW) as < 750 kDa.

**Results:**

HMW IAHA was associated with a statistically significant and possibly clinically significant improvement in pain (SMD − 0.57 (95% credible interval [Crl]: − 1.04, − 0.11), exceeding the − 0.50 MCII threshold. LMW IAHA had a lesser, non-significant improvement (− 0.23, 95% Crl: − 0.67, 0.20). Back-transforming SMDs to the WOMAC pain scale indicated a 14.65 (95% CI: 13.93, 15.62) point improvement over IA placebo, substantially better than the 8.3 AAOS MCII threshold.

**Conclusions:**

Unlike LMW IAHA, HMW IAHA exceeded the MCII threshold for pain relief, suggesting that improvements can be subjectively perceived by the treated patient. Amalgamation of LMW and HMW may have blurred the benefits of IAHA in the past, leading to negative recommendations. Differentiation according to MW offers refined insight for treatment with IAHA.

**Supplementary information:**

**Supplementary information** accompanies this paper at 10.1186/s12891-020-03729-w.

## Background

A common approach for managing osteoarthritis (OA) is by attenuating inflammation and pain in affected joints [1]. Some theorized mechanisms for the therapeutic effect of viscosupplementation through intraarticular hyaluronic acids (IAHAs) include possible anti-inflammatory properties and chondroprotection by mitigating proteoglycan loss in cartilage and apoptosis of chondrocytes [2, 3]. IAHA treatment has also been shown to stimulate endogenous production of additional HA by human synoviocytes suggesting IAHA may restore viscoelastic properties to the synovial fluid in patients with OA [2].

IAHAs possess a wide range of possible molecular weights (MW) [4–8]. IAHAs have been consistently labeled as high molecular weight (HMW), moderate molecular weight (MMW), and low molecular weight (LMW). Although the nomenclature has been consistently used, defining thresholds have varied [4–7]. HMW has been used to refer to molecules ranging from ≥3000 kDa [4] to ≥6000 kDa [5, 7]. MMW has been defined as MW ranges of > 1500 to < 3000 kDa [4], ≥ 1500 to < 6000 kDa [7], and 800 to 2000 kDa [5]. LMW has been defined as ≤1500 kDa [4], < 1500 kDa [7], and 500 to 730 kDa [5]. Heterogeneity in MW thresholds within each sub-class across the literature can be attributed to varying reasons, including trial investigators who focus on specific products or others who seek to validate treatment effects observed in broader MW ranges [4, 8]. Despite this variability, consistent patterns in therapeutic differences have been reported with higher MW IAHA products showing superior efficacy over lower MW IAHA products [4–8].

In 2013, the American Academy of Orthopaedic Surgeons (AAOS) published an update to their 2008 clinical practice guidelines (CPGs) for patients with knee OA [9]. The ninth recommendation was against the use of IAHA for patients with symptomatic knee osteoarthritis on the basis of 14 studies, of which three were designated as “high-quality”. Since this recommendation included two or more high-quality studies, the overall recommendation was labeled “Strong”. This decision was primarily focused on lack of efficacy; specifically, that IAHA effect sizes did not meet minimal clinically important improvement (MCII) thresholds. Though reviewers noted significant benefits associated with HMW IAHAs, the lack of significance between other IAHA MWs resulted in grouping IAHAs as a single category [4].

Following the findings of AAOS, which indicated differences in treatment effects between HMW and LMW, and the availability of new evidence published since 2013, we conducted an updated systematic review and network meta-analysis (NMA) to examine the relative efficacy of HMW IAHAs compared with LMW IAHAs, intraarticular (IA) placebo, and other non-surgical FDA-approved treatments. For consistency, inclusion/exclusion criteria developed by AAOS in their 2013 CPG update were adopted without alteration for the current investigation. This study also evaluated whether the effect size of HMW IAHA was greater than accepted thresholds for MCII.

## Methods

### Systematic review

As this review was a replication and update of the AAOS 2013 systematic review and meta-analysis, all criteria from the original publication were carried over into the current study [9].

#### Search strategy

Literature searches were executed on November 1, 2018 within MEDLINE (via PubMed), Embase (via Ovid), and Cochrane Central for all randomized controlled trials (RCTs) in humans published since 1966 or database inception. Search strategies included strings with MeSH terms and key words for “hyaluronic acid,” “knee,” “osteoarthritis,” and “randomized controlled trial.” Language (English) and subject (humans) limits were applied (Additional file 1).

The relevance of each article was determined by two, independent investigators at title/abstract and full-text stages using criteria based on the AAOS CPG (Table 1**)**. Any disagreement was resolved by a third investigator. Screening was conducted on DOC Library (Doctor Evidence LLC, Santa Monica, CA, US).

**Table 1:** AAOS inclusion/exclusion criteria [9]

Inclusion Criteria	Exclusion Criteria
• Study was of osteoarthritis of the knee • Study reported on 80% of the patient population of interest • Article provided full report of a clinical study • Study was published in a peer-reviewed journal • Study had a sample of 30 or more patients per treatment group • Study was of humans • Study was published in English • Study was published during or after 1966 • Study results were presented quantitatively • Study treatment follow up period was at least 4 weeks • At least 80% of the enrolled study population were 19 years of age or older • For any included study that used “paper-and-pencil” outcome measures (e.g. SF-36), only those that were validated were included [unless the outcome was identified a priori by the work group in the critical outcomes Delphi round]	• Retrospective non-comparative case series, medical records review, meeting abstracts, historical articles, editorials, letters, and commentaries • Case series studies that gave patients the treatment of interest AND another treatment • Case series studies that had non-consecutive enrollment of patients were excluded • Controlled trials in which patients were not stochastically assigned to groups AND in which there was heterogeneity in patient characteristics or outcomes at baseline AND where the authors did not statistically adjust for these differences when analyzing the results • All studies of “Very Limited” evidence strength • Composite measures or outcomes even if they were patient-oriented • Case series studies with no baseline values • “Paper and pencil” outcomes (e.g. SF-36) reported by a single group of investigators • Study performed on cadavers • In vitro study

*SF-36* 36-Item Short Form Survey

#### Inclusion and exclusion criteria

A prespecified protocol based on the Population, Intervention, Comparator, and Outcome (PICO) framework was developed to guide the review methodology (Table 2). Briefly, all RCTs evaluating non-surgical, FDA-approved therapies for knee OA were included. HMW IAHAs were defined as products whose MW was at least 6000 kDa, which is the most mimetic to endogenous HA within synovial fluid [10]. In keeping with the AAOS CPG categorizations of IAHAs, LMW IAHAs were considered those less than 750 kDa. Publications on comparisons of HMW vs HMW, LMW vs LMW, or only investigating products whose MWs were between 750 and 6000 kDa were excluded. Pain scores were the primary outcomes of interest. A comprehensive list of specific scales/scores assessing pain included in this review are presented in Table 2.

**Table 2:** PICO criteria

PICO	Inclusion Criteria
Participants	• Persons with knee OA
Interventions	• Intraarticular corticosteroids o Triamcinolone o Methylprednisolone o Betamethasone o Triamcinolone Sustained Release • Intraarticular Hyaluronic Acids • Non-Steroidal Anti-Inflammatory Drugs (NSAIDs) o Diclofenac o Celecoxib o Naproxen • Acetaminophen • Oral Opioids
Comparators	• Any
Outcomes	• Efficacy (pain hierarchy from highest to lowest priority) o WOMAC pain subscale (Likert; 0–20) (VAS; 0–500) o Pain during activity (VAS; 0–100) o Pain during walking (VAS; 0–100) o Global knee pain (VAS; 0–100) o Pain at rest (VAS; 0–100) o SF-36 (bodily pain (BP) subscale; 0–100) o HAQ (pain subscale; 0–100/0–3), Lequesne algofunctional index (pain subscale; 0–8), AIMS (pain subscale; 0–10), Knee-Specific Pain Scale (KSPS), McGill Pain Questionnaire (pain intensity; 0–50), ASES (pain subscale; 10–100), SES (Schmerzempfindungsskala – pain perception scale) o Pain at night (VAS; 0–100), pain during activity (NRS; 0–10), pain on walking (NRS; 0–10), number of painful days (days)

*AIMS* Arthritis Impact Measurement Scale; *ASES* Arthritis Self-Efficacy Scale; *HAQ* Health Assessment Questionnaire; *NRS* numerical rating scale; *OA* osteoarthritis; *SF-36* 36-Item Short Form Survey; *VAS* Visual Analogue Scale; *WOMAC* Western Ontario and McMaster Universities Arthritis Index

#### Data configuration

Two investigators performed data configuration for each accepted article using DOC Data 2.0 (Doctor Evidence LLC, Santa Monica, CA, US). Discrepancies were resolved by a third investigator. Trial, patient, and treatment characteristics along with pain outcomes were extracted for each included article based on PICO. The mean, median, variance, and ranges were extracted for all continuous pain scores across the studies. Instances in which a publication presented data points of interest in graphs or charts without explicit mention of the values displayed were captured using Grab-It software (Datatrend, Minnetonka, MN, US).

#### Quality assessment

Quality assessments were performed to determine the risk of bias within each publication by two investigators. The methods are described in detail in the 2013 AAOS CPG [9]. In brief, a coding scheme was used which consists of incremental increases for the following domains: adequate statistical power, random assignment of patients to comparison groups, sufficient blinding, comparability of the patient groups at study start, integrity of treatment delivery (to make sure the observed differences between the groups could reasonably be attributed to the treatment), use of validated outcome measures, and the absence of investigator bias. Studies were considered to be High, Moderate, Low, or Very Low quality if there were 0, 1–2, 3–4, or > 5 domains, respectively, which were “flawed”. Quality assessments performed as part of the original systematic review by AAOS were included without manipulation.

### Statistical analysis

#### Treatment classifications

A Bayesian NMA of indirect treatment comparisons was performed using five treatment categories: HMW IAHAs, LMW IAHAs, IA corticosteroids, conventional therapy, and IA placebo. The level of network connectivity was first determined to assess the feasibility of conducting an analysis using these categories. Once a connected network was confirmed, an investigation into the most relevant effect measures was performed.

#### Primary endpoint

Pain scores were collected at 1, 3, 6, 9, and 12-months follow-up; however, the longest follow-up timepoint was used in the NMA when multiple timepoints were reported. A single effect size from each publication was selected to create a robust network. In publications where more than one variation of pain outcomes were available, the hierarchy in Table 3 was used to determine which effect size to include in the NMA [11]. Mean differences between interventions and comparators were converted to standardized mean differences (SMDs) before being incorporated into the NMA to adjust for the heterogeneity in pain scales reported across the evidence base. SMD was calculated and then run using a normal/identity link-likelihood model. Details on the statistical packages and models used are provided in Additional file 2.

**Table 3:** Hierarchy of pain measures

Rank	Measure
1	WOMAC pain sub-scale (Likert: 0–20; VAS: 0–500)
2	Pain during activity (VAS: 0–100)
3	Pain during walking (VAS: 0–100)
4	Global knee pain (VAS: 0–100)
5	Pain at rest (VAS: 0–100)
6	SF-36 bodily pain (BP) sub-scale (0–100)
7	HAQ pain sub-scale (0–100; 0–3), Lequesne algofunctional index pain sub-scale (0–8), AIMS pain sub-scale (0–10) Knee-Specific Pain Scale (KSPS) McGill Pain Questionnaire – pain intensity (0–50) ASES pain sub-scale (10–100) Schmerzempfindungsskala (SES) Pain Perception Scale
8	Pain at night (VAS: 0–100), Pain during activity (NRS: 0–10), Pain on walking (NRS:0–10), Number of painful days (days)

*AIMS* Arthritis Impact Measurement Scales; *ASES* American Shoulder and Elbow Surgeons Shoulder Score; *HAQ* Health Assessment Questionnaire; *NRS* numeric rating scale; *SF-36* 36-Item Short Form Survey; *WOMAC* Western Ontario and McMaster Universities Osteoarthritis Index; *VAS* visual analog scale

#### Overall rank probabilities

In each iteration of the Markov chain Monte Carlo simulation (part of the Bayesian framework), the treatments were ranked according to effect size. The rank probability is the percent of the time that each treatment was first, second, third and so on in this ranking [12].

#### Minimally Important Clinical Improvements & Statistical Significance

**Fig. 1:**
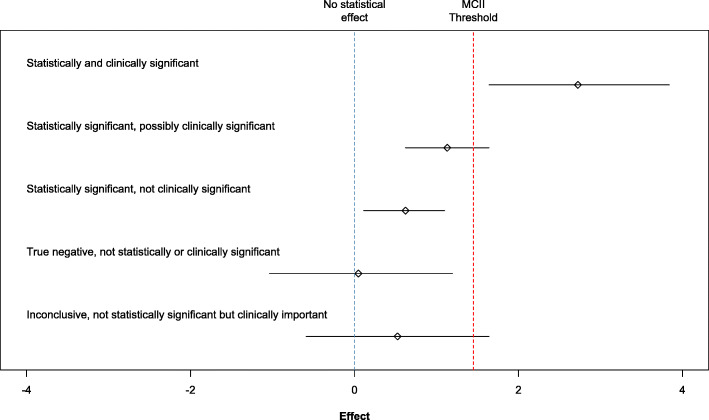
Confidence intervals of treatment effects that range in statistical and clinical significance adapted from the 2013 American Academy of Orthopaedic Surgeons clinical practice guidelines [9]. Footnote: MCII = minimum clinically important improvement

Criteria for determining clinical significance for results with MCII were adapted from Armitage et al. 2001 [13, 14] and described in detail in the AAOS CPG [9]. For reference, pain assessments conducted using the WOMAC required treatment effect sizes greater than 0.50 and absolute improvements of − 8.3 relative to placebo to be considered clinically significant. AAOS required a smaller effect size (− 0.39) for their MCII threshold, which is encompassed in our more stringent threshold of − 0.50. This threshold was lowered in keeping with more conservative criteria published since the time of the original review [15]. As a crude guideline, effect sizes between 0.30 and 0.50 are considered to be minimally clinically or subjectively important. A visual depiction of clinical and statistical significance can be found in Fig. 1 [9]. Results were considered statistically significant if *p* < 0.05.

## Results

### Systematic review

A total of 628 articles were retrieved as a result of all literature searches conducted (Additional file 3**)**. Following de-duplication, 404 unique records were screened at the title/abstract stage of which 208 records were excluded. The remaining 196 publications were assessed for eligibility at the full-text stage, of which 146 publications met PICO eligibility criteria. However, for the purposes of analyses in the NMA only 14 studies reporting on 2796 patients were suitable for a connected network [16–29].

The evidence base for the NMA was comprised of literature deemed to be of moderate (*n* = 9) [18–22, 24, 26–28] or high quality (*n* = 5) [16, 17, 23, 25, 29]. Each publication was sufficiently powered for the evaluation of pain through WOMAC or VAS scores, and free from flaws within timepoint measurements. Quality assessment details can be found in Additional file 4.

Details on study-level and patient characteristics, outcomes, and timepoints can be found in the Additional files 5, 6 and 7. WOMAC scores were incorporated into the NMA from nearly half of the literature [16, 18, 21, 23, 24, 29]. Effect sizes were measured between 2-month (*n* = 1) [19] and 12-month (*n* = 3) [17, 20, 24] study follow-up, with the most common timepoint measurement reported at 6 months (*n* = 7) [18, 22, 23, 25, 26, 28, 29].

Baseline WOMAC pain across trials and treatment arms using a 0–20 scale ranged from 8.8 to 13.9 [16, 22, 24, 28], across trials using a 0–100 scale ranged from 45.39 to 50.3 [21, 29], and across the one trial using a 0–4 scale it was 2.3 and 2.38 respectively for the two treatment groups [18]. OA severity/grading was measured mainly using the Kellegren-Lawrence grading scale across five trials [21, 23–25, 29]. The proportion of patients across trials and treatment arms with Grade I ranged from 10.7 to 24%, Grade II ranged from 12.5 to 60%, Grade III ranged from 36.9 to 61%, and Grade IV ranged from 14 to 38.8%. Radiographic disease severity was assessed in two trials and was measured by the Larsen Grade [26, 27]. Proportion of patients across trials and treatment arms with severities of Grade I, II, III, and IV ranged from 7 to 16%, 37–56%, 25–47%, and 3–11%, respectively [26, 27]. Other baseline measures of pain and OA severity are captured in Additional file 5.

### Network-meta analysis

**Fig. 2:**
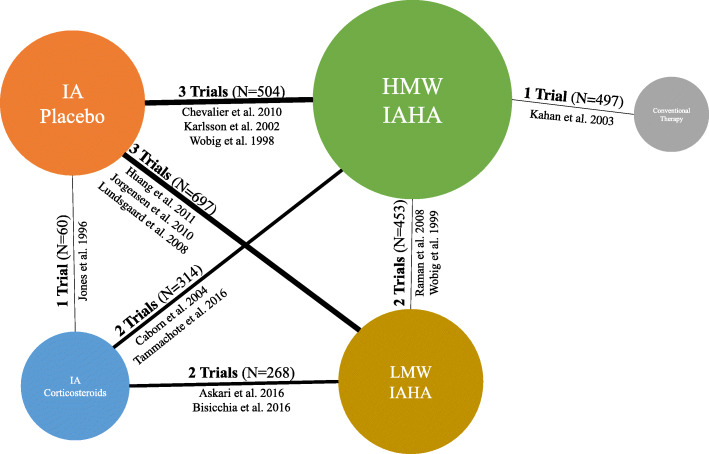
Network of trials comparing improvement in pain scores in patients with knee osteoarthritis receiving nonoperative treatments. Footnote: The size of the nodes is proportional to the total number of participants. HMW = high molecular weight; LMW = low molecular weight; IA = intraarticular; IAHA = intraarticular hyaluronic acid

The network included five nodes of interest: HMW IAHAs, LMW IAHAs, IA corticosteroids, conventional therapy, and IA placebo (Fig. 2). Though trials investigating non-steroidal anti-inflammatory drugs (NSAIDs), acetaminophen, and oral opioids were relevant, they were excluded from the analysis because they lacked a common comparator. Fourteen data points from 14 studies were incorporated into the NMA. Residual deviance was calculated to be 1.041 and I^2^ was 11%.

**Fig. 3:**
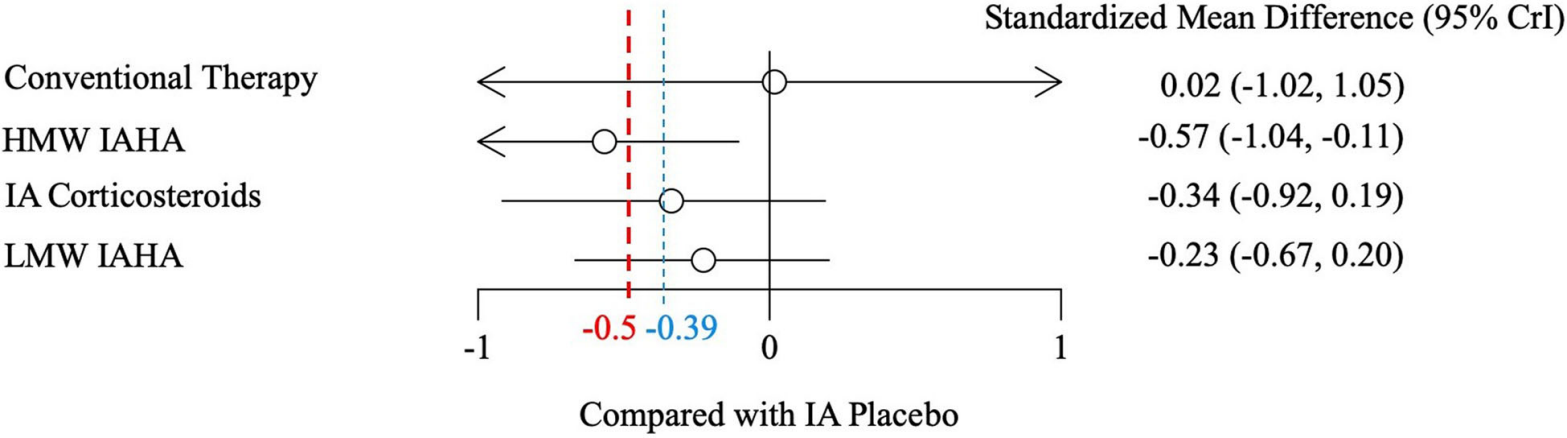
Standardized mean differences (and 95% credible intervals) for each intervention node compared to intraarticular placebo. Footnote: Crl = credible interval; HMW = high molecular weight; IA = intraarticular; IAHA = intraarticular hyaluronic acid; LMW = low molecular weight; MCII = minimal clinically important improvement. Blue dotted line indicates MCII threshold used by AAOS CPG [9]. Red dotted line indicates conservative MCII threshold used in the current analysis as established by Angst et al. 2017 [15]

Improvements in pain scores relative to IA placebo can be found in Fig. 3. HMW IAHA demonstrated a statistically significant mean difference of − 0.57 (95% Crl: − 1.04, − 0.11) compared to IA placebo, and considered “possibly clinically significant” according to the threshold used by the AAOS [9]. HMW IAHA continued to demonstrate greater improvement in pain than all other intervention categories, including LMW IAHA (SMD -0.34 [95% Crl: − 0.82, 0.13]), but none of these differences were significant (Table 4). LMW IAHA (− 0.23 [95% Crl: − 0.67, 0.20]) and IA corticosteroids (− 0.34 [95% Crl: − 0.92, 0.19]) had greater improvements in pain than IA placebo; these differences were not statistically significant, but clinically important.

**Table 4:** League table

HMW IAHA	IA Corticosteroids	LMW IAHA	Conventional Therapy	IA Placebo
**HMW IAHA**	−0.23 (− 0.72, 0.30)	−0.34 (− 0.82, 0.13)	−0.59 (− 1.52, 0.34)	***−0.57 (− 1.04, − 0.11)***
	**IA Corticosteroids**	−0.11 (− 0.66, 0.39)	−0.36 (− 1.44, 0.72)	−0.34 (− 0.92, 0.19)
		**LMW IAHA**	−0.25 (− 1.27, 0.82)	− 0.23 (− 0.67, 0.20)
			**Conventional Therapy**	0.02 (−1.02, 1.05)
				**IA Placebo**

Each cell represents the comparison (SMDs and 95% CrI) of the row versus the column interventions. Italicized values are statistically significant. Negative values favor the row treatment. *HMW* high molecular weight; *IA* intraarticular; *IAHA* intraarticular hyaluronic acid; *LMW* low molecular weight

**Fig. 4:**
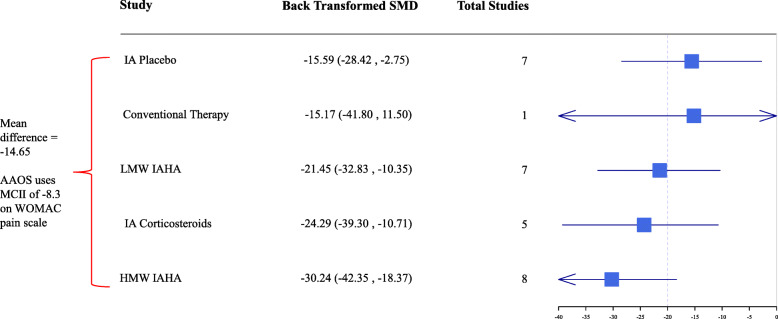
Absolute efficacy on WOMAC pain 0–100 scale. Footnote: SMDs were multiplied by median SD (25.8) to convert effect estimates back to WOMAC Pain Scale (0–100). AAOS = American Academy of Orthopaedic Surgeons; HMW = high molecular weight; IA = intraarticular; IAHA = intraarticular hyaluronic acid; LMW = low molecular weight; MCII = minimal clinically important improvement; SMD = standardized mean difference; WOMAC = Western Ontario and McMaster Universities Arthritis Index

**Fig. 5:**
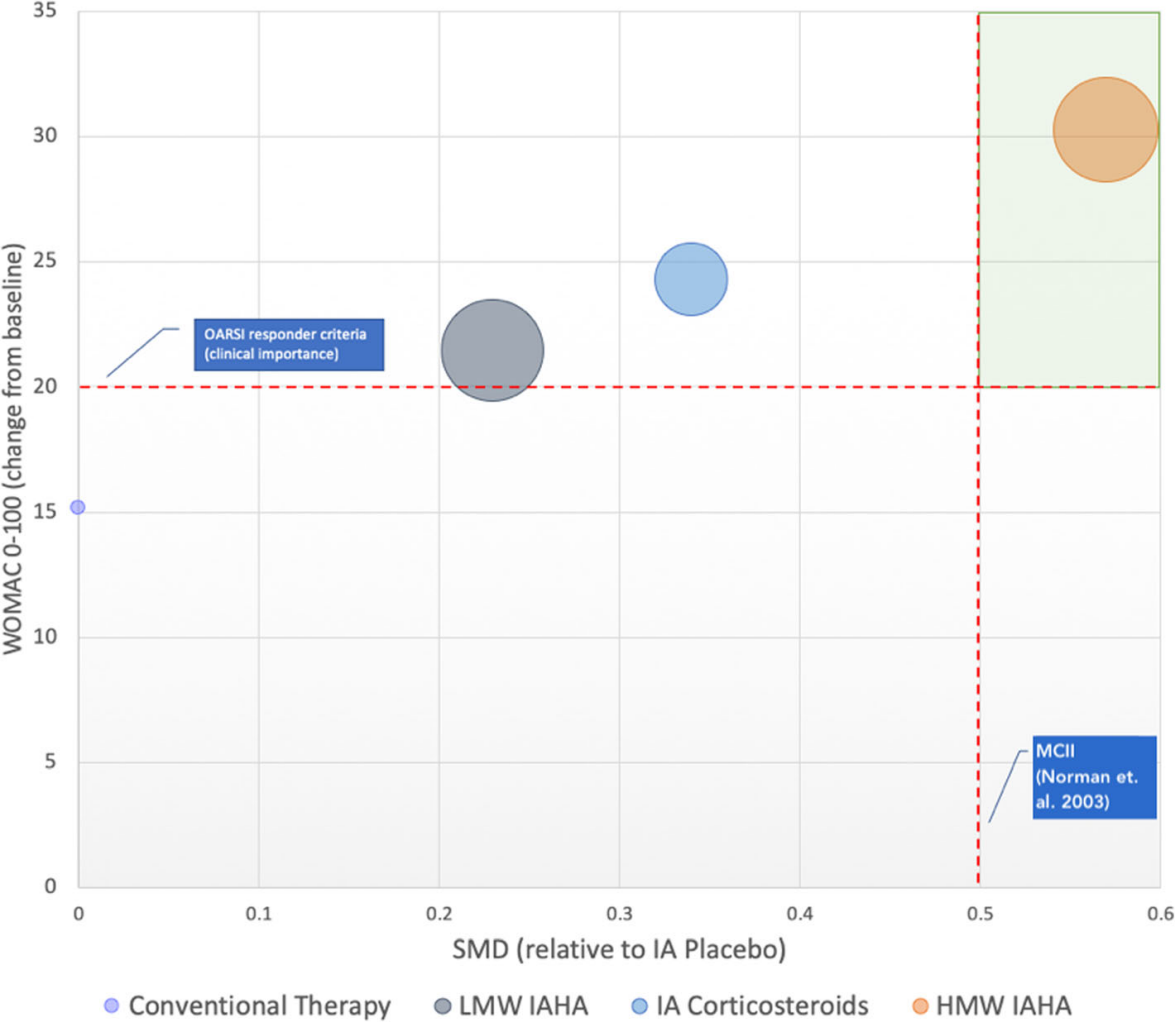
Cluster graph showing absolute efficacy (change from baseline on WOMAC 0–100 scale) plotted against relative efficacy (compared to IA Placebo). Footnote: HMW = high molecular weight; IA = intraarticular; IAHA = intraarticular hyaluronic acid; LMW = low molecular weight; SMD = standardized mean difference; WOMAC = Western Ontario and McMaster Universities Arthritis Index

SMDs converted to WOMAC Pain scale (0–100) provide additional evidence of absolute differences in effect sizes between treatment categories (Fig. 4). In comparison with IA placebo, HMW IAHA achieved a greater improvement in pain scores exceeding MCII thresholds (MD: − 14.65). The cluster graph in Fig. 5 demonstrates HMW IAHA exceeded the MCII threshold of 0.5 and exceeded the Osteoarthritis Research Society International responder criteria for clinical importance (change ≥20 points on WOMAC pain scale) [30]. By comparison, LMW IAHA versus IA placebo did not meet MCII.

**Fig. 6:**
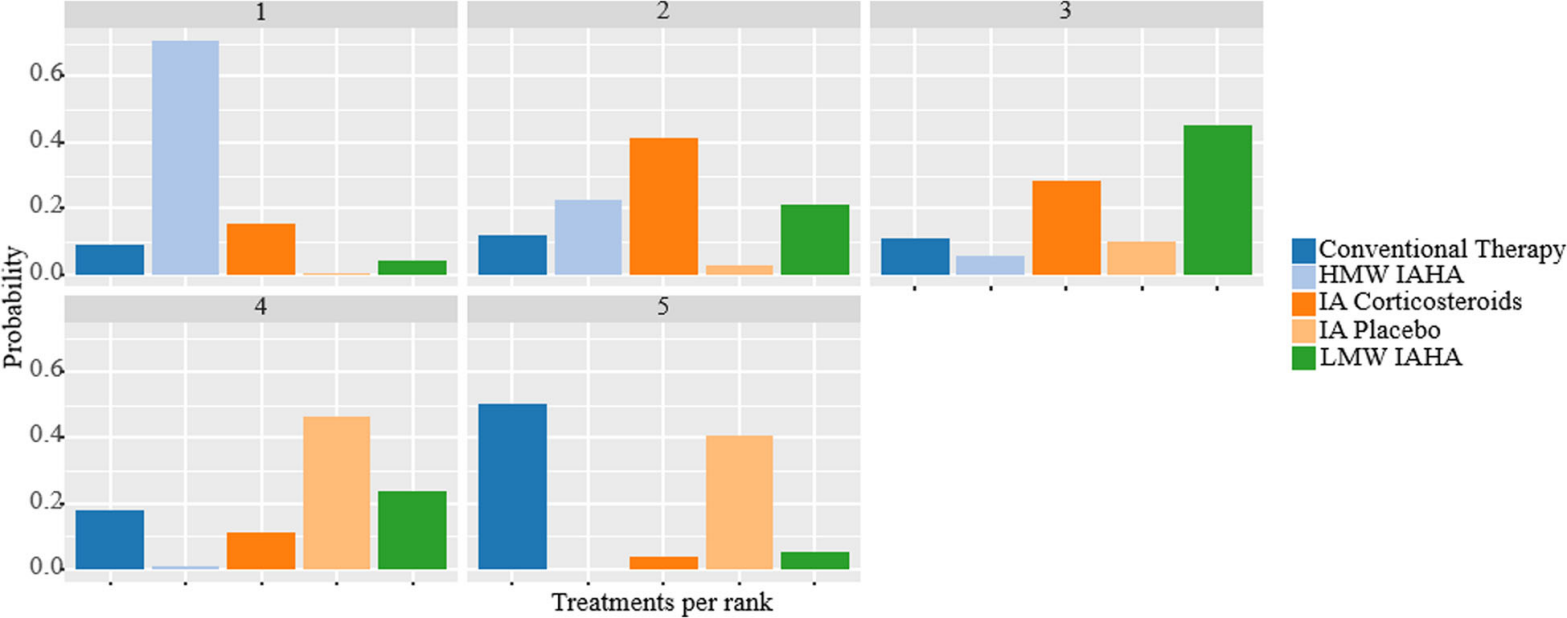
Rankogram. Footnote: HMW = high molecular weight; IA = intraarticular; IAHA = intraarticular hyaluronic acid; LMW = low molecular weight

Overall rank probabilities are provided in Table 5 and Fig. 6. The highest likelihood of first-rank was HMW IAHA. IA corticosteroids, LMW IAHA, IA placebo, and conventional therapy were positioned as second, third, fourth, and fifth positions based on their respective probabilities for each ranking.

**Table 5:** Probability of treatment being a certain rank

Treatment	Rank 1	Rank 2	Rank 3	Rank 4	Rank 5
HMW IAHA	**71.05**	22.55	5.50	0.90	0.00
IA Corticosteroids	15.55	41.20	28.20	11.20	3.85
Conventional Therapy	8.85	12.00	10.95	18.05	50.15
LMW IAHA	4.32	21.32	45.15	23.60	5.60
IA Placebo	0.22	2.93	10.20	46.25	40.40

^a^*HMW* high molecular weight; *IA* intraarticular; *IAHA* intraarticular hyaluronic acid; *LMW* low molecular weight

Additional figures for node splitting results displaying mean differences between treatments using direct and indirect comparisons can be found in Additional file 8. There were no significant differences between the mean differences found from direct and indirect comparisons. Analysis of heterogeneity can be found in Additional file 9. The I^2^ of direct comparisons ranged from 41 to 93.3% and 0 to 90.8% for indirect comparisons.

## Discussion

This network meta-analysis highlights the possible clinical significance of HMW IAHAs as a treatment for patients with knee osteoarthritis. HMW IAHAs demonstrated superiority over IA placebo in terms of statistical and possible clinical significance. By comparison, LMW IAHAs and IA corticosteroids demonstrated greater improvements in pain scores; however, neither intervention class was statistically significant when compared against IA placebo. Although safety was not the focus of this review, we observed that 10 out of the 14 included studies conducted safety assessments in addition to efficacy, and nine studies reported no significant differences between the treatment groups with regard to adverse events.

All evidence included in the analysis was obtained from RCTs and deemed moderate-to-high in quality using the AAOS criteria. RCTs are considered highly within this appraisal framework, which is a major contributing factor in elevating the level of quality of the body of literature. Of note, all 14 studies demonstrated sufficient power in their measurement of specific pain outcomes.

Our results challenge the practice of broadly grouping IAHAs into a single intervention node as was found in the supporting evidence for AAOS CPGs [9]. When HMW IAHAs are split into a separate treatment category, a notable distinction in therapeutic benefits is observed from other drug classes, including LMW IAHAs. HMW IAHAs were statistically significantly superior to placebo, whereas LMW IAHAs were not. In fact, our analysis demonstrated greater improvements in pain management with HMW IAHAs relative to LMW IAHAs, though the differences between HMW versus LMW did not prove statistically significant nor did they exceed the thresholds for MCII. Interestingly, AAOS authors acknowledged a similar distinction among IAHA’s sub-classes in that most statistically significant outcomes in their analysis were associated with HMW IAHAs [9]. MMW IAHAs were previously found to be similar to LMW IAHAs by the AAOS, and thus were not relevant for the purposes of this investigation [9]. Though, it is noteworthy that some trials have studied the treatment patterns of MMW IAHAs relative to HMW and LMW categories [5, 6, 31–33].

HMW IAHAs have previously demonstrated superiority to other interventions, including IAHAs of other MWs [1, 4, 7, 34]. In a recent systematic review on pharmacological interventions and medical devices, HMW IAHAs (≥6000 kDa) were found superior to 19 other treatments for knee OA [34]. In fact, the reported effect sizes surpassed strict MCII thresholds (0.50) for HMW and MMW (< 6000 and ≥ 1500 kDa) IAHAs after adjusting for IA placebo effects (0.29) [34]. LMW IAHAs (< 1500 kDa) were considered “possibly clinically important” in that analysis [34]. Altman et al. 2016 also examined intra-class differences of IAHAs using WOMAC pain scores as the primary efficacy measure [4]. Though thresholds for HMW, MMW, and LMW varied slightly from those in our categorization strategy, results from their analysis are consistent with ours, demonstrating a relationship between increasing MW and improvement in pain outcomes. Similarly in another study, distinctions between LMW IAHAs, HMW IAHAs, and very HMW IAHAs showed a progression of increasing benefit for pain measurements [7]. In both studies, larger IAHA molecules demonstrated superiority statistically using established MCII standards (− 0.37 effect sizes) [4, 7]. MCII standards for absolute reductions in this analysis were set at − 0.50 to conform to a stricter threshold than that reported by AAOS, as previously described elsewhere (range: − 0.50 to − 0.37) [15, 35]. Further, SMDs converted back to WOMAC scores demonstrated a benefit surpassing MCII standards used by AAOS CPGs (effect size < − 8.3) [9]. Interestingly, this threshold has been recently updated to improve the generalizability of other patients with knee OA assessed on WOMAC pain criteria [36]. Unlike the former threshold, the updated SMD (effect size < − 7.09) on the WOMAC pain scale for improvement is specific to knee OA and incorporates adjustments for sex, age, and WOMAC baseline pain. The therapeutic benefits of HMW IAHAs are even more pronounced with this new criterion.

### Strengths and limitations

This updated systematic review and network meta-analysis provides additional insights into the distinction that should be made with regard to molecular weight whenever evaluating comparative treatment effects across multiple IAHAs. The analysis presented here is supported by an evidence base that has largely addressed issues of bias within each publication. Though a complete network with all five nodes of interest was feasible, some comparisons between nodes in the network were challenged by a paucity of literature. For instance, connections between IA corticosteroids and IA placebo [19], and HMW IAHAs and conventional therapy [21], were formed through a single study in each comparison. In the latter case, this publication was the only linkage to the larger network diagram. Similarly, other treatment categories relevant to this patient population were excluded from the network due to a lack of common comparators linking them to the network. For this reason, only 14 articles were included in the NMA of the eligible 146 articles included in the systematic review.

## Conclusion

HMW IAHAs offer a competitive advantage in reducing pain symptoms for patients with knee OA. The therapeutic benefits of this sub-class of IAHAs met standards for statistical significance and are possibly clinically meaningful. Additional research is needed to strengthen direct and indirect comparisons found within this network diagram, and to incorporate additional treatment categories. Namely, it will be important for future trials to investigate how the treatment categories investigated in this analysis relate to NSAIDs, oral opioids, and acetaminophen.

## Supplementary information

**Additional file 1.** Search Strategies.

**Additional file 2.** Statistical Packages and Models.

**Additional file 3.** PRISMA Diagram.

**Additional file 4.** Quality assessment.

**Additional file 5.** Included studies characteristics and outcome data.

**Additional file 6.** Included studies and timepoints used.

**Additional file 7.** Number of studies per outcome.

**Additional file 8.** Node splitting results.

**Additional file 9.** Analysis of heterogeneity (ANOHE).

## Data Availability

Not applicable. The data used for analysis was retrieved from openly published studies listed in our manuscript.

## Abbreviations

HMW: High molecular weight
MMW: Moderate molecular weight
LMW: Low molecular weight
IAHA: Intraarticular hyaluronic acids
OA: Osteoarthritis
NMA: Network meta-analysis
IA: Intraarticular
MCII: Minimal clinically important improvement
AAOS: AMERICAN Academy of Orthopaedic Surgeons
CPGs: Clinical practice guidelines
SMDs: Standardized mean differences
