# Supplementary material for: High molecular weight Intraarticular hyaluronic acid for the treatment of knee osteoarthritis: a network meta-analysis
Source: BMC Musculoskelet Disord. 2020 Oct 23;21:702. doi: 10.1186/s12891-020-03729-w (PMC7585216; doi:10.1186/s12891-020-03729-w)
Supplement: Supplementary file 2 — Additional file 2. Statistical Packages and Models. [file 12891_2020_3729_MOESM2_ESM.docx]

## **Additional file 2: Statistical Packages and Models.**

A random-effects model was run using the R package “geMTC”. This package utilizes JAGS, a program for Bayesian models using the Markov Chain Monte Carlo simulation. A burn-in of 5,000 for 2 chains and 20,000 additional iterations were run with a thinning parameter of 10. The deviance information criterion was 27.33 for the random-effects model and 27.45 for the fixed-effects model, supporting the decision to use the random effects model. To assess the efficacy based on the Western Ontario and McMaster Universities Osteoarthritis Index (WOMAC) 0-100 scale, back transformation was conducted by multiplying SMDs via Hedge’s g by the median standard deviation (SD) of 25.8 of the oral placebo group, which was the most reported group that measured pain on the 0-100 scale. Heterogeneity was assessed using I^2^ and p<0.05 was considered statistically significant.
