# Supplementary material for: High molecular weight Intraarticular hyaluronic acid for the treatment of knee osteoarthritis: a network meta-analysis
Source: BMC Musculoskelet Disord. 2020 Oct 23;21:702. doi: 10.1186/s12891-020-03729-w (PMC7585216; doi:10.1186/s12891-020-03729-w)
Supplement: Supplementary file 3 — Additional file 3. PRISMA Diagram. [file 12891_2020_3729_MOESM3_ESM.docx]

## **Additional file 3: PRISMA Diagram.**

Trials not eligible to be included in the NMA

(n = 132)

Unique records screened based on title and abstract, after de-duplication

(n = 404)

Additional records identified through Bannuru publication

(n = 137)

Records identified through database searching

(n = 491)

**Included**

**Eligibility**

**Screening**

**Identification**

Trials met eligibility criteria

(n = 146)

Trials included

in NMA

(n = 14)

Full-text articles excluded, with reasons (n = 50)

Wrong comparison (n = 26)

Wrong population (n = 6)

Wrong study design (n = 2)

Wrong outcome (n = 6)

Non-English (n = 1)

Missing outcome (n = 1)

Duplicate publication (n = 6)

Abstract with insufficient information (n = 1)

Wrong intervention (n = 1)

Full-text articles assessed for eligibility

(n = 196)

Records excluded

(n = 208)
