# Supplementary figures and images for: High molecular weight Intraarticular hyaluronic acid for the treatment of knee osteoarthritis: a network meta-analysis
Source: BMC Musculoskelet Disord. 2020 Oct 23;21:702. doi: 10.1186/s12891-020-03729-w (PMC7585216; doi:10.1186/s12891-020-03729-w)

## **Additional file 6: Included studies and timepoints used.**


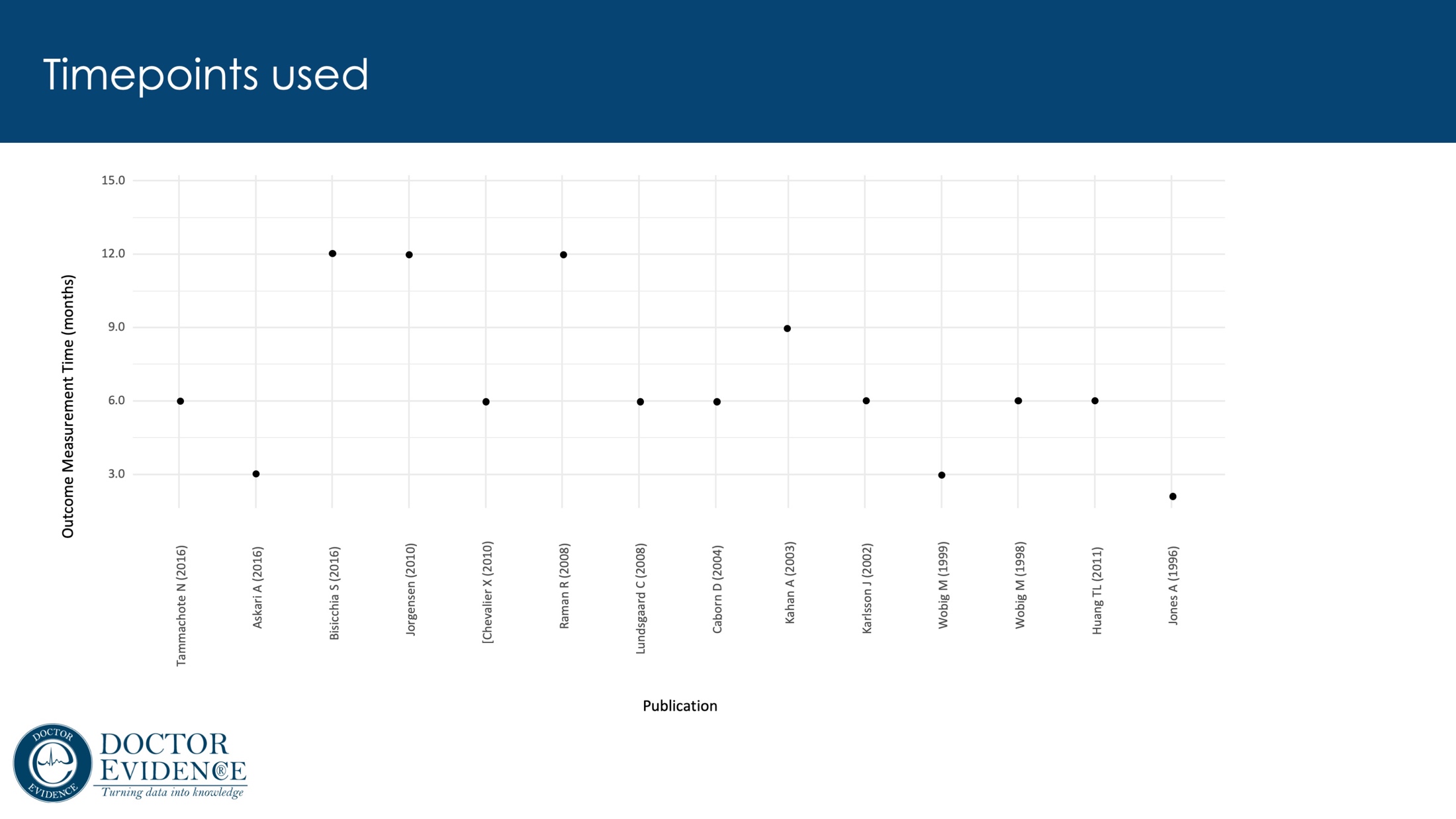

Supplement: Supplementary file 6 — Additional file 6. Included studies and timepoints used. [file 12891_2020_3729_MOESM6_ESM.docx]
