# Supplementary material for: High molecular weight Intraarticular hyaluronic acid for the treatment of knee osteoarthritis: a network meta-analysis
Source: BMC Musculoskelet Disord. 2020 Oct 23;21:702. doi: 10.1186/s12891-020-03729-w (PMC7585216; doi:10.1186/s12891-020-03729-w)
Supplement: Supplementary file 8 — Additional file 8. Node splitting results. [file 12891_2020_3729_MOESM8_ESM.docx]

## **Additional file 8: Node splitting results.**


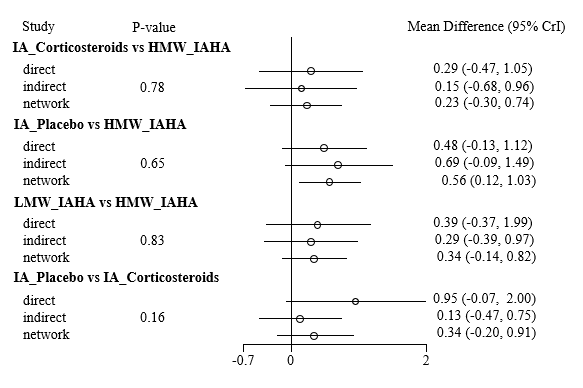


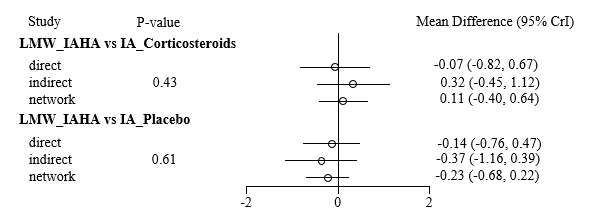


Crl = credible interval; HMW = high molecular weight; IA = intraarticular; IAHA = intraarticular hyaluronic acid; LMW = low molecular weight
